# Responding to health and social needs of aging Latinos in new-growth communities: a qualitative study

**DOI:** 10.1186/s12913-017-2551-2

**Published:** 2017-08-25

**Authors:** Kim Larson, Holly F. Mathews, Essie Torres, C. Suzanne Lea

**Affiliations:** 1 College of Nursing, East Carolina University, 3135 Health Science Building, Greenville, NC 27858 USA; 2 Department of Anthropology, East Carolina University, Greenville, NC 27858 USA; 3 Department of Health Education and Promotion, East Carolina University, Greenville, NC 27858 USA; 4 Department of Public Health, Brody School of Medicine, East Carolina University, Greenville, NC 27858 USA

**Keywords:** Latinos, New-growth community, Interprofessional, qualitative description

## Abstract

**Background:**

The development of new-growth communities of Latino immigrants in southern states has challenged the traditional health and social service infrastructure. An interprofessional team of service providers, Latino leaders, and university faculty partnered to establish linkages with the Latino community and providers serving aging adults and to explore the health and social needs of aging Latinos residing in a rural region.

**Methods:**

A qualitative descriptive study was conducted through a community-university partnership, the Aging Latino Research Team (ALRT). Data were generated from nine focus groups and 15 key informant interviews with Latino and non-Latino community members and service providers in rural, eastern North Carolina (ENC).

**Results:**

Thematic analysis was used to identify common patterns and form recommendations for future research and programs. Themes common to Latino participants were: “We are put off to one side”; “If I can't work, I can't survive”; and “Without documents, you are no one.” Themes common to non-Latino participants were: “Older Latinos are not well served”; “Older Latinos are invisible”; “Older Latinos are undocumented and afraid”; and “Older Latinos are wandering the highway”.

**Conclusion:**

A major finding of this research was the extent to which discrepancies in perceptions between Latino participants and non-Latino participants exist. These discrepancies revealed ethnic stereotyping and cultural insensitivity as major barriers in access to care.

## Background

The Latino population in the United States of America (US) is increasing in size, visibility, and diversity. Projections are that Latinos aged 65 and older will increase from the current 8% of the US population to 22% by 2060 [1, 2]. Data support that aging Latinos are more likely to have limited formal education, lower income, and poorer health outcomes than the general population [3, 4]. Adversity over the life course, punctuated by low incomes and few assets, results in economic insecurity in old age [5].

Historically, Latino populations have been concentrated in California, Texas, Florida, and New York [6]. Latino resettlement across the US has generated new-growth communities especially in the Southeast, Midwest, and Northeast [7–9]. New-growth communities are those with an emergent and growing concentration of Latinos in areas with no previously established population [7]. Several studies have reported on the health status of Latinos in new-growth communities in the Midwest and Northeast, but these studies have focused on young Latino men or urban Latino residents.

The Southeastern region leads the US in Latino population growth [10, 11] and North Carolina’s (NC) Latino population increased by 111% between 2000 and 2010 [12]. In 2014, an estimated 890,000 Latinos resided in NC, of which 11% (97,000) were age 50 years and older [13] compared to 9% nationally [14]. No studies were found that focused on the health and social service needs of aging Latinos in new-growth communities in the rural southeastern US, such as those found in NC. The purpose of this study was two-fold: to establish linkages with the Latino community and providers serving aging adults and to describe the health and social needs of aging Latinos in rural eastern NC (ENC). For this study, the aging Latino population was defined as age 50 and older, specifically immigrants from Mexico and Central America, who have chosen to reside in NC. The majority of services for seniors in North Carolina begin at age 55; using age 50 captures the number of Latinos approaching eligibility for purposes of strategic planning.

### Contextual significance

In NC, 33.6% of Latinos live below the federal poverty level compared to 17.2% in the general population, and are three times more likely to be uninsured than non-Hispanic Whites [15]. Although the leading causes of death among Latinos in NC are similar to the general population, such as cancer, heart disease and stroke, the prevalence of risk factors for these chronic conditions is disproportionately higher among Latinos [16]. A recent study on immigration policies and the Latino population in NC has identified infrastructure weaknesses, such as lack of qualified interpreters, public transportation in rural areas, and primary care providers [9].

Three studies evaluated health and social service needs of Latinos in new-growth communities in the Midwest and Northeast. In the Midwest, Lanesskog et al. [17] conducted interviews with 25 professionals predominately from the education sector, with equal numbers of non-Latino and Latino participants. The four factors that assisted service delivery to Latinos were language competence, cultural knowledge in multiple contexts, empathy toward clients, and the will to act [17]. A study in the Northeast used a community-based participatory research approach to assess the health needs of immigrant Latino men [18]. Data were generated from four focus groups with Latino men and interviews with 10 health and social service staff. The three themes reported were social isolation, staying healthy, and accessing the health care system [18]. The third study was a needs assessment conducted in Baltimore, Maryland, and among the five focus groups only one included aging Latino women [19]. The top health concerns of this group were “bone-related disease,” hypertension and high cholesterol, stress (*nervios*), respiratory problems, diabetes, influenza, and AIDS. Barriers reported by all study participants were linguistic, cultural, financial, legal, and logistical (transportation and communication).

We framed our study on various theoretical views and findings that emphasize interpersonal linkages and cultural attributes salient to health care utilization and access barriers among aging Latinos [20, 21]. This modified framework featured macro upstream factors including socio-demographic characteristics, social determinants of health, and health and social service outcomes.

## Methods

This qualitative descriptive study was conducted by the Aging Latino Research Team (ALRT), a community-university partnership between the Eastern Carolina Council Area Agency on Aging (AAA) and East Carolina University (Greenville, North Carolina). Key administrators from the AAA, a Latino leader from the target region, and faculty from Anthropology, Health Education and Promotion, Nursing, Public Health, and Sociology comprised this interprofessional, bilingual research team. The ALRT members designed structured interview guides using language and cultural concepts relevant for the Latino population. Six university students in nursing, public health, and anthropology assisted the research team. This study focused on three target counties, Duplin, Greene, and Wayne, in ENC, all of which have Latino populations (11% - 22%), exceeding the statewide average (9%) [22]. The university institutional review board approved the study. A waiver of signed informed consent was granted and verbal consent was obtained from all participants.

### Sample and setting

Service providers from eight ENC counties were recruited by the key administrator from the Eastern Carolina Council AAA. Focus groups were planned in conjunction with a scheduled regional meeting and conducted after the meeting for the convenience of participants. Twenty-six individuals participated in one of four focus groups held concurrently at the AAA regional office. These participants included staff from AAA, senior centers, social services and health departments, Spanish interpreters and an immigration attorney. The majority (92.3%) of provider focus group participants were white females.

Participants for Latino focus groups were recruited through local churches and community leaders in the three target counties. Thirty-nine Latino community members aged 50 and older participated in five focus groups. Among the five Latino focus groups, both men (28.2%) and women (71.7%) were represented. The majority of participants were from Mexico and fewer were from Honduras, Guatemala, and El Salvador. The focus group demographics are presented in Table 1.

**Table 1:** Focus Group Demographics

Focus Groups	Provider Focus Groups (PFG) *N* = 4				Latino Focus Groups (LFG) *N* = 5				
	PFG1	PFG2	PFG3	PFG4	LFG1	LFG2	LFG3	LFG4	LFG5
	*n* = 6	*n* = 6	*n* = 7	*n* = 7	*n* = 4	*n* = 10	*n* = 3	*n* = 8	*n* = 14
Sex									
Female	4	6	7	7	3	7	3	3	12
Male	2	0	0	0	1	3	0	5	2
Race/Ethnic Background									
White	4	4	5	4	0	0	0	0	0
Black	1	1	1	3	0	0	0	0	0
Latino	1	1	1	0	4	10	3	8	14
Target Counties Represented									
Duplin	X			X		X			
Greene		X	X	X					X
Wayne	X		X	X	X		X	X	
Other	X	X	X	X					

Fifteen key informants from the three target counties were identified through snowball sampling. Key informants were known to focus group or research team members as having had long-term contact with Latinos in their communities. These included male (40%) and female (60%), Latino (53%) and non-Latino (47%) service providers, business professionals, community advocates, faith-based community members, and Latino leaders.

### Data collection

Data were collected between October 2015 and June 2016. The three data sets were: a) service provider focus groups, b) Latino community member focus groups, and c) key informants (Latino and non-Latino). All interviews and focus groups were audio-taped, followed a structured interview guide (available on-line as supplemental material), lasted approximately 30–90 min, and were transcribed within several days after the meeting date. Research team members moderated and co-moderated all focus groups and conducted all key informant interviews. Interviews took place at convenient community locations and in the preferred language of participants, either English or Spanish. Spanish transcripts were translated by a native speaker into English. Participants received either a gift card or pad-folio for their contribution to the study.

### Data management and analysis

Focus group and key informant transcripts were read independently by all research team members. The research team met monthly to discuss coding strategies and come to consensus on findings. A matrix was created of the focus group transcripts organized by interview question to identify key concepts and facilitate between case and within case comparisons. The ALRT members had on-going discussions to determine commonalities and differences. A constant comparison approach was used to identify patterns in the data and discover relationships between ideas or concepts [23]. Transcripts from key informant interviews were analyzed in two ways. First, a content analysis [24] was performed to tabulate answers to the interview questions and compare these across participants looking for common patterns. Next, transcripts were analyzed following a Grounded Theory framework to identify broader themes emblematic of attitudes shared by a group [25, 26]. Triangulation between the three data sets was used to discover commonalities in health problems, barriers to care, and health perceptions until data saturation was achieved.

## Results

Major health issues facing aging Latinos in order of frequency are described in Table 2. Chronic diseases, particularly diabetes, heart disease and hypertension, were reported as the top health concerns of aging Latinos by all focus groups. The acute health problems reported were heat stress and work-related injuries, broken bones, and respiratory infections. Mental health issues reported included dementia, Alzheimer’s disease, depression, stress, and insomnia. Additional complaints were obesity, poor nutrition, and problems with hearing, vision, and dental care. Two culture-bound syndromes reported were *susto* (fright) and *nervios* (nerves). Only Latino focus group participants emphasized musculoskeletal problems, such as arthritis.

**Table 2:** Major health issues for aging Latinos

Chronic Disease and Chronic Disease Management Issues:	
1. Diabetes	
2. Heart Disease	
3. Hypertension	
4. High cholesterol	
5. Arthritis	
6. Rheumatism	
7. Asthma	
8. Bronchitis	
9. Cancer	
Problems Secondary to Poor Diabetes Self-Management:	
1. Vision problems	
2. Limb/ft problems	
3. Kidney disease	
4. Liver disease	
Mental Health Issues:	
1. Dementia	
2. Alzheimer’s disease	
3. Depression	
4. Stress	
5. Insomnia	
Acute Health Issues:	
1. Heat Stress	
2. Work-related injuries; broken bones	
3. Sexually transmitted infections	
4. Respiratory infections	
Other Health Issues:	
1. Alcoholism (especially among men)	
2. Obesity and poor nutrition	
3. Bone/joint problems	
4. Dental problems	
5. Hearing issues	
Culture-bound Syndromes: *susto* and *nervios*	

Perceived barriers to health and social service programs at the individual and systems levels are displayed in Table 3. We note that some barriers could be relevant at both levels.

**Table 3:** Perceived barriers to access health care for aging Latinos

Individual level:	
• Inability to speak English	
• Low educational levels	
• Lack of health literacy	
• Lack of private transportation	
• Inability to obtain a driver’s license	
• Low incomes	
• Lack of health insurance	
• Undocumented status and inability to qualify for services	
• Lack of knowledge about eligibility requirements for different services	
• Lack of telephones making patient follow-up difficult	
• Lack of family support	
Systems level:	
• Limited hours of operation at health facilities	
• Shortage of primary care providers	
• Long delays in obtaining appointments	
• Lack of Spanish-speaking personnel in health agencies	
• Discrimination due to lack of cultural sensitivity	
• Lack of public transportation in rural areas	
• Undocumented status and inability to qualify for services	
• Lack of information in Spanish on services available to aging adults	
• Expensive fees for service	
• Agencies with limited budgets must target state and federal priorities	
• Ongoing budget cuts limit hiring of bilingual staff or translation services	
• Competition at the local level between agencies for resources	
• Lack of community partnerships to assist Latinos	

### Individual barriers

Inability to speak English and low levels of formal education were major barriers for many aging Latinos. Low wages and lack of health insurance were related to the types of employment available to immigrant workers. Aging Latinos without legal status lacked access to some services, while others were unsure about eligibility requirements. Lack of family support and social isolation in rural ENC also impacted health outcomes dramatically.

### System barriers

Most health and human services agencies in the target counties lacked Spanish-speaking interpreters often due to insufficient funding. One service provider focus group reported that a social service agency serving older adults had recently adopted an “English-only” workplace policy. Bilingual interpreters were often shared by multiple agencies and Latino advocacy groups in the target counties were absent. Service providers reported lack of funding and interagency competition for limited resources. Further noted was a lack of community partnerships or efforts to collaborate to find mutual solutions to service needs and programmatic gaps.

Aging Latinos experienced discrimination in the health care system from both providers and staff members, even when they themselves were Latino. For example, Latinos waited longer than other clients to be seen by providers. Latinos also perceived that they were treated rudely, looked down upon for lack of formal education and undocumented immigration status, and were rushed through their appointments. Public transportation in rural areas was either absent or not available to undocumented residents, who also had difficulty obtaining an official driver’s license due to state policies.

A shortage of primary care providers contributed to the long delays in obtaining appointments. Some agencies also had limited hours of operation, and fees for service were sometimes costly, further limiting accessibility. Latino focus group participants reported the use of various strategies to manage costs such as not purchasing full supplies of medicines, not taking the full amount prescribed, borrowing medicines from other people, using home remedies and consulting with alternative practitioners such as herbalists and *curanderos* (natural healers).

The eligibility criteria for some social services were unclear for aging Latinos. Differences in eligibility requirements were noted by county and funding source. For example, eligibility for services offered by AAA, such as medication assistance, in-home care, and home meal deliveries, varied between counties. Yet, even when eligible, some aging Latinos reported being turned away due to either lack of clarity about eligibility criteria by service providers or absence of interpreters. Aging Latinos were also afraid to seek out services due to fear of deportation as a result of local law enforcement collaborating with the federal Immigration and Customs Enforcement agency.

Thematic analysis revealed key differences between Latino and non-Latino participants. These are discussed below and labeled with a quotation that encapsulates the meaning behind each theme. Three themes characterized the perceptions of Latino participants. The first theme was “We are put off to the side.” Latino participants felt they were discriminated against by health and service providers because of their ethnicity and lack of English language skills. Participants reported feeling as if their needs were put aside. For example, participants reported waiting for services only to find out no help was available. They perceived that they did not have a voice. As one person articulated, “Even the receptionist at the window will not attend to us the same as they attend to others.”

The second theme was “If I can’t work, I can’t survive.” Participants emphasized that aging Latinos can never retire. If they become sick or disabled, aging Latinos have no government support. Since the majority work at hard physical labor, ill health, particularly diseases that impact work ability, are a major problem. For example, one person reported having trouble working and managing his diabetes, while another recounted how arthritis and knee pain caused him to lose his job and become homeless.

The third theme was “Without documents, you are no one.” Participants pointed out that Latinos arrive from many different countries; some on temporary visas, some have permanent residency, but others are undocumented residents. The undocumented residents are often discriminated against even within the Latino community.

Four themes characterized the perceptions of non-Latino participants. The first theme was “Older Latinos are not served well.” Non-Latino participants agreed that public agencies in these counties did not serve Latinos well due to the lack of Spanish language and cultural skills training among staff. When staff members treat Latinos poorly, they do not return. Providers were aware and recognized this issue although providers also noted it was sometimes due to situational factors not intent. As one person articulated, “When physicians and nurses are driven by the need to see a lot of people in a short time and it takes more than twice as long to evaluate a Latino patient, they cut corners [when interacting with the Latino patient].”

The second theme was “Older Latinos are invisible.” Non-Latino participants discussed how aging Latinos live with their families and are hidden from public view. As one person said, “You do not see them on the streets very often or out and about.” The assumption among non-Latino participants was this invisibility is a positive sign because the Latino culture is family-oriented and families willingly provide care for Latino elders.

The third theme was “Older Latinos are undocumented and afraid.” Non-Latino participants assumed that aging Latinos are undocumented and are afraid to access services because they fear deportation. Further, they saw aging Latinos as unwilling to seek out information on services or attend community events because of these fears. For these same reasons they are unable to drive or get to services. As one person replied, “…changes need to come from above; the political system, before we can do anything.”

The fourth theme was “Older Latinos are wandering the highway.” Non-Latino participants observed occurrences of aging Latinos wandering in the community, lost and confused. There are few mental health facilities for aging Latinos making referrals and care challenging. One service provider was informed of a working Latino family who left their aging relative alone during day and he often went missing.

## Discussion

The key findings from our study of aging Latinos in a new-growth community support both those reported for new migrant populations and long-established Latino communities. Similar to other recent migrant populations engaged primarily in farm work, our participants reported a range of work-related musculoskeletal problems and occupational-related injuries. Yet providers and Latinos also listed chronic diseases similar to those experienced by longer-established communities [7, 19, 27]. Unique to our sample was the identification of dementia and related disorders by service providers.

The focus groups and key informants identified several chronic conditions perceived to be increasing among aging Latinos that are similar to those found in other studies [19, 28]. These conditions include diabetes, heart disease, hypertension and high cholesterol. Participants also placed emphasis on arthritis and other musculoskeletal problems which have not been as widely reported in other studies. Cheriel et al. [29] note that the burden of arthritis in the US is minimized by providers due to underutilization of best practice interventions, lack of access to care, and cultural and language barriers.

Latino participants in this study, like those in other studies of new growth communities [19, 30] also listed mental health concerns including depression, stress and anxiety as problems they experienced but for which they had not sought treatment. Additionally, two culture-bound syndromes of *susto* (fright) and *nervios* (physical and/or mental unrest) were reported. Both present with symptoms of distress; however, *nervios* is related more with continual stresses, while *susto* has been related to a single stressful event [31]. Baer et al. [31] reported that *nervios* was not perceived as a mental illness by a sample of Mexican-American and Guatemalan farmworkers. In our study, participants referred to *susto* and *nervios* in response to the anxieties and stresses experienced with immigration and fears of deportation. These findings confirm results from the National Latino and Asian American Study by Alegria et al. [32], which found that US born Latinos have higher rates of mental disorders, particularly anxiety, depression and substance abuse, than do more recent immigrants. A review of service use found that Latinos underutilize mental health services and rely on primary care providers making them less likely to receive congruent care [33]. It would seem of great importance for clinicians working with these populations to understand how the terms *nervios* and *susto* (and possibly other culture-bound syndromes) are used, and further investigate their relationship to stress, depression, physiological symptoms, and other mental health conditions.

A unique finding of this study was the discrepancy found in perceptions between Latino and non-Latino participants regarding access to care, familism, and mental health. Latino participants viewed unnecessary long clinic waits, poor customer service, and lack of concern by providers as discrimination. This finding aligns with research showing that 30% of Latinos nationwide believe that racism is a major problem in health care and that 58% are concerned about being treated unfairly due to race/ethnicity when seeking care [34].

In contrast, service providers believed Latino clients were not treated well due to situational factors, such as not taking time to determine eligibility, lack of interpreters, and lack of cultural competence. Addressing this discrepancy is as critical as eliminating access barriers because when Latinos are treated poorly, they tend to not return for healthcare. This leads to missed appointments, lack of trust, lack of information, and underutilization of preventive health and social services that may result in adverse outcomes [28].

Familism was also perceived differently by these two groups. Latino participants expressed the need to work and not depend on family members for financial and caregiving support. Service providers assumed that Latino families did not want to use government services and that they preferred to care for their elders. There is limited research on family support in immigrant communities; however, two studies suggest that Chinese and Korean elders receive less family support in the US and perceive they must depend more upon themselves [35, 36]. The implication of this assumption by service providers is that Latino families are doing an adequate job and do not need or want help for aging family members. Although some aging Latinos are cared for by family members, providers should not assume relatives are able to do so. One solution would be to assess aging Latinos for independence in activities of daily living and refer families to appropriate resources.

While Latino participants did not verbalize dementia as a major health condition, the service providers noted incidents of aging Latinos lost and confused with potential signs of dementia. These three target counties lack dementia care facilities or home respite care. Therefore, working Latino families are challenged to provide care for affected relatives. Our findings are consistent with those of Gonzalez, Haan and Hinton [37] who documented an overall dementia prevalence of 31% among their sample of Latinos in California age 80 and over. These investigators also reported 43% of dementia was directly attributable to type 2 diabetes mellitus, stroke or a combination of the two. These findings suggest that the high prevalence of diabetes among Latinos in ENC may lead to increasing prevalence of dementia as this population ages. An effective public health approach to address this looming problem is to focus on primary and secondary prevention of diabetes among younger Latinos.

Our study also confirms common access barriers in other studies that focus on Latino new-growth communities in general. These barriers include language [19, 30, 38, 39], cultural insensitivity [18, 19, 39], lack of interpreters [19, 39], transportation [19, 38, 39], immigration issues [19, 38], lack of insurance [19, 28, 38, 39], and cost of care [18, 19, 30, 39]. An important finding unique to our study is the legitimate fear that aging Latinos in new-growth communities in ENC have of deportation due to racial profiling and harassment. Undocumented status in the US offers no form of security, provides limited civil and labor rights, and creates a significant barrier for immigrant integration into communities [40]. Increasingly, certain laws have made it easier to shift from documented to undocumented status (i.e. H-2a temporary agricultural workers program) but not vice versa, placing many immigrants in legal limbo for indefinite periods of time [41]. This shift has led to more extensive law enforcement strategies, which heightens the risk for deportation, immigration raids, and civil rights violations among undocumented community members [40]. In ENC, these enforcement strategies have been reported to include a variety of public spaces, such as churches, community clinics, public bus stops, and traffic stops. Therefore, these experiences have negative consequences for daily lives of Latino community members in new-growth communities, and are a critical barrier in access and use of health and social services. It is important to note that legal status intensifies the effects of disadvantages, and has intergenerational impacts on communities [40], which can potentially further exacerbate existing health disparities. Future research needs to examine the intergenerational effect of legal status on health within a family and community context.

### Strengths and limitations

The strengths of this study were the use of purposive sampling to recruit both service providers and Latino community members, which allowed for identification of overlapping perceptions from different viewpoints. The key informants represented a wide variety of community leaders. These samples also generated rich data from focus groups and key informants. One limitation was that the provider sample was predominantly white and female, reflecting provider demographics in the Eastern Carolina Council AAA. This racial and gender bias may impede a full understanding of Latino cultural issues. Still, respondents recognized this limitation in the data. The study was also limited by the current fears prevalent among undocumented Latinos, which restricted the collection of detailed demographic data on Latino focus group participants.

## Conclusions

This is the first study to demonstrate discrepancies in perceptions among Latino and non-Latino participants, and the potential implications these attitudes can have on service delivery at various levels. These discrepancies may contribute to missed clinical and community-based opportunities to empower and intervene with families to thwart some of the potential intergenerational health effects of discrimination and lack of access to needed health and social services. This study also strongly suggests that service providers in new-growth, and largely rural, communities could benefit from cultural sensitivity training. Service providers with the Eastern Carolina Council AAA programs are incorporating the study’s findings into their current strategic plans.

## Abbreviations

AAA: Area agency on aging
ALRT: Aging Latino research team
ENC: Eastern North Carolina
LFG: Latino focus groups
PFG: Provider focus groups
